# Development of a panel of three multiplex allele-specific qRT-PCR assays for quick differentiation of recombinant variants and Omicron subvariants of SARS-CoV-2

**DOI:** 10.3389/fcimb.2022.953027

**Published:** 2022-08-18

**Authors:** Jianguo Li, Zefeng Gao, Jing Chen, Ruiling Cheng, Jiahui Niu, Jialei Zhang, You Yang, Ximei Yuan, Juan Xia, Guoli Mao, Hulong Liu, Yongkang Dong, Changxin Wu

**Affiliations:** ^1^ Shanxi Provincial Key Laboratory of Medical Molecular Cell Biology, Institutes of Biomedical Sciences, Shanxi University, Taiyuan, China; ^2^ Shanxi Provincial Key Laboratory for Major Infectious Disease Response, Shanxi Provincial Center for Disease Control and Prevention, Taiyuan, China; ^3^ College of Life Sciences, Shanxi University, Taiyuan, China; ^4^ Laboratory, Shanxi Guoxin Caregeno Biotechnology Co., Ltd, Taiyuan, China; ^5^ Administrative Office, the Fourth People's Hospital of Taiyuan, Taiyuan, China

**Keywords:** SARS-CoV-2, allele-specific qRT-PCR, mutation, recombinant variant, Omicron variant, BA.1 subvariant, BA.2 subvariant

## Abstract

Quick differentiation of the circulating variants and the emerging recombinant variants of SARS-CoV-2 is essential to monitor their transmission. However, the widely used gene sequencing method is time-consuming and costly when facing the viral recombinant variants, because partial or whole genome sequencing is required. Allele-specific real time RT-PCR (qRT-PCR) represents a quick and cost-effective method in SNP genotyping and has been successfully applied for SARS-CoV-2 variant screening. In the present study, we developed a panel of 3 multiplex allele-specific qRT-PCR assays targeting 12 key differential mutations for quick differentiation of SARS-CoV-2 recombinant variants (XD and XE) and Omicron subvariants (BA.1 and BA.2). Two parallel multiplex qRT-PCR reactions were designed to separately target the protype allele and the mutated allele of the four mutations in each allele-specific qRT-PCR assay. The variation of Cp values (ΔCp) between the two multiplex qRT-PCR reactions was applied for mutation determination. The developed multiplex allele-specific qRT-PCR assays exhibited outstanding analytical sensitivities (with limits of detection [LoDs] of 2.97-27.43 copies per reaction), wide linear detection ranges (10^7^-10^0^ copies per reaction), good amplification efficiencies (82% to 95%), good reproducibility (Coefficient of Variations (CVs) < 5% in both intra-assay and inter-assay tests) and clinical performances (99.5%-100% consistency with Sanger sequencing). The developed multiplex allele-specific qRT-PCR assays in this study provide an alternative tool for quick differentiation of SARS-CoV-2 recombinant variants (XD and XE) and Omicron subvariants (BA.1 and BA.2).

## Introduction

The current circulating Omicron variant of SARS-CoV-2 has diverged into at least three subvariants (BA.1, BA.2 and BA.3) (Desingu and Nagarajan, 2022). Dozens of mutations were observed in the Omicron variants, among which at least 6 mutations were unique in the BA.1 subvariant (NSP3: L1266I; NSP6: I189V; Spike: S371L, G446S, T547K, and L981F) (Yu et al., 2022), and at least 6 mutations were unique in the BA.2 subvariant (NSP1: S135R, NSP3: T24I, NSP6: F108L, Spike: A27S, V213G, R408S) (Majumdar and Sarkar, 2022). The BA.2 subvariant is becoming the predominant strain of SARS-CoV-2 by replacing the BA.1 subvariant with a higher transmissibility (Chen and Wei, 2022). It is reported that BA.2 could potentially evade natural and vaccine-induced immunity, rather than BA.1 (Bartsch et al., 2022). The discrepancies in transmissibility and pathogenicity between the BA.1 and BA.2 subvariants make it necessary to conduct differential diagnosis of the Omicron subvariants in COVID-19 patients (Hamill et al., 2021).

Along with the predominant circulation of the Omicron variants, several recombinant variants of SARS-CoV-2 have emerged (He et al., 2022; UK Health Security Agency, 2022; Wertheim et al., 2022). Among the Omicron-recombinant variants (UK Health Security Agency, 2022), the XD variant is from recombination of the Delta variant and the Omicron BA.1 subvariant at the site nt 21643 (numbered as the prototype strain NC_045512.2, the same hereafter) and the site nt 25581; the XE variant is a recombinant variant between the Omicron BA.1 and BA.2 subvariants at the site nt 11537; the XF variant is a recombinant between the Delta variant and the Omicron BA.1 subvariant at the site nt 5386. Besides the Omicron-recombinant variants, Alpha-Epsilon (Wertheim et al., 2022) and Beta-Delta (He et al., 2022) recombinant variants were also reported. Although the pathogenicity and transmissibility of the SARS-CoV-2 recombinant subvariants remain largely undetermined and required further evaluation, previous reports have suggested elevated transmissibility and enhanced pathogenicity of the recombinant coronaviruses (Phillips et al., 1999; Su et al., 2016). Quick differentiation approaches are required to monitor the transmission of SARS-CoV-2 recombinant variants.

While gene sequencing (Sanger sequencing or Next Generation Sequencing) is a commonly applied strategy for viral variant identification (Srivastava et al., 2021), differentiation of massive recombinant variants is time and cost-consuming because of requiring partial or whole viral genome sequencing. Allele-specific real time RT-PCR (qRT-PCR) integrate the advantages of real-time PCR and allele-specific PCR, representing a highly sensitive and time-saving method for SNP genotyping (Gaudet et al., 2009). Allele-specific qRT-PCR has been successfully applied for identification of SARS-CoV-2 mutations, exhibiting a promising potential in differentiation of viral variants (Graber et al., 2021; Wang et al., 2021).

In the present study, we developed a panel of three multiplex allele-specific qRT-PCR assays, targeting 12 key mutations for differentiation of the Omicron BA.1 and BA.2 subvariants and the recombinant XD and XE variants of SARS-CoV-2.

## Materials and methods

### Ethical statement

The use of desensitized clinical specimens for evaluation of the developed allele-specific qRT-PCR assays was approved by the Ethics Committee of Shanxi University with a project identification code SXULL2022035.

### Viral genome sequence analysis and primer/probe design

A total of 1,093,239 genome sequence of SARS-CoV-2 with high quality (< 1% Ns) deposited in GISAID (https://www.gisaid.org) were retrieved. Sequence alignment was performed by using the Muscle algorithm implemented in MEGA v7.0 (Kumar et al., 2016). The key mutations in Omicron BA.1 and BA.2 subvariants (**Table 1**) were confirmed and viral genomic regions suitable for primer/probe design were selected with the help of Primer Express v3.0 (Applied Biosystems, CA, USA). Primers and probe were designed according to the following principles: choosing regions with GC content of 40-60%; an amplicon of 75-250bp; avoiding regions with secondary structure; avoiding regions with long (> 4) repeats of single bases; avoiding the guanine (G) at the 5’ end of probes; all the selected primers have similar Tm (between 59°C and 61°C) and Tm of the corresponding probes were 5-10°C higher.

**Table 1:** Primers & probes for the developed multiplex allele-specific qRT-PCR assays.

Assay		Allele	Forward primer (5’-3’)	Probe (5’-3’)	Reverse primer (5’-3’)	Amplicon (bp)
Prototype allele targeting reactions	Assay 1	NSP1: S135R	TAATAAAGGAGCTGGTGGCCATTGT	FAM-CGGCGCCGATCTAAAGTCATTTGACTTAGGCGAC-BHQ1	CCACAGAAGTTGTTATCGACATAGCG	194
		NSP6: I189V	CTGTCATGTTTTTGGCCAGAGCTA	VIC-TTGGCCTCTTTTGTTTACTCAACCGCTACTTTAGACTGACTC-BHQ1	GTGGGAGTAGTCCCTGTGAATTC	242
		Spike: A27S	CCAGAACTCAATTACCCCGTG	Cy3-CTTGGTTCCATGCTATACATGTCTCTGGGACCAATGGT-BHQ2	CCATCATTAAATGGTAGGACAGGGTTATC	207
		Spike: S371L	CTGTGTTGCTGATTATTCTGTCCTATATAAATC	Cy5-AGGTGATGAAGTCAGACAAATCGCTCCAGGGC-BHQ2	GTAAATTGCGGACATACTTATCGGC	179
	Assay 2	NSP6: F108L	GGATATGGTTGATACTAGTTTGTCTGGATT	FAM-CCTTATGACAGCAAGAACTGTGTATGATGATGGTGCTAGGA BHQ1	GGAAATGGCTTGATCTAAAGCATTACC	195
		Spike: G446S	CTTGGAATTCTAACAATCTTGATTCTAAGGATG	VIC-CAACTGAAATCTATCAGGCCGGTAGCACACCTTGT BHQ1	CATTAGTGGGTTGGAAACCATATGATTGT	197
		Spike: T547K	ACAAATGTGTCAATTTCAACTTCAATGGTTTTAC	Cy3-CTGCCTTTCCAACAATTTGGCAGAGACATTGCTGACAC-BHQ2	ATGGTGTAATGTCAAGAATCTCAAGTGTC	161
		Spike: L981F	TTGGTGCAATTTCAAGTGTTTTAAATGATAACC	Cy5-CTTTCACGTCTTGACAAAGTTGAGGCTGAAGTGCA-BHQ2	TTGATTGTCCAAGTACACACTCTGAC	203
	Assay 3	NSP3: T24I	GTGCAAGGTTACAAGAGTGTGAATATGAC	FAM-TGAGAAGTGCTCTGCCTATACAGTTGAACTCGGTACAGAAG BHQ1	GGTGTAAGTAATTCAGATACTGGTTGCAAAG	178
		NSP3: L1266I	GTAGGAGACATTATACTTAAACCAGCAAATAATACTT	VIC-CAGAAGAGGTTGGCCACACAGATCTAATGGCTGC BHQ1	GGGTTTTCAAACCTAATACTCTAGATAATTCATTAGG	150
		Spike: V213G	AATATATTCTAAGCACACGCCTATTAATTTTGT	Cy3-CGTGATCTCCCTCAGGGTTTTTCGGCTTTAGAACC-BHQ2	CACCAGGAGTCAAATAACTTCTATGTAAAGC	151
		Spike: R408S	GATTCATTTGTAATTAGAGGTGATGAAGTCTGA	Cy5-CGCTCCAGGGCAAACTGGAAAGATTGCTG-BHQ2	GGTAATTATAATTACCACCAACCTTAGAATCAAG	162
Mutated allele targeting reactions	Assay 1	NSP1: S135R	TAATAAAGGAGCTGGTGGCCATTGG	FAM-CGGCGCCGATCTAAAGTCATTTGACTTAGGCGAC BHQ1	CCACAGAAGTTGTTATCGACATAGCG	194
		NSP6: I189V	TGTCATGTTTTTGGCCAGAGCTG	VIC-TTGGCCTCTTTTGTTTACTCAACCGCTACTTTAGACTGACTC BHQ1	GTGGGAGTAGTCCCTGTGAATTC	241
		Spike: A27S	AACCAGAACTCAATTACCCCGTT	Cy3-CTTGGTTCCATGCTATACATGTCTCTGGGACCAATGGT-BHQ2	CCATCATTAAATGGTAGGACAGGGTTATC	209
		Spike: S371L	CTGTGTTGCTGATTATTCTGTCCTATATAAACT	Cy5-AGGTGATGAAGTCAGACAAATCGCTCCAGGGC-BHQ2	GTAAATTGCGGACATACTTATCGGC	179
	Assay 2	NSP6: F108L	GGATATGGTTGATACTAGTTTGTCTGGATA	FAM-CCTTATGACAGCAAGAACTGTGTATGATGATGGTGCTAGGA BHQ1	GGAAATGGCTTGATCTAAAGCATTACC	195
		Spike: G446S	CTTGGAATTCTAACAATCTTGATTCTAAGGATA	VIC-CAACTGAAATCTATCAGGCCGGTAGCACACCTTGT BHQ1	CATTAGTGGGTTGGAAACCATATGATTGT	197
		Spike: T547K	ACAAATGTGTCAATTTCAACTTCAATGGTTTTAA	Cy3-CTGCCTTTCCAACAATTTGGCAGAGACATTGCTGACAC-BHQ2	ATGGTGTAATGTCAAGAATCTCAAGTGTC	161
		Spike: L981F	TTGGTGCAATTTCAAGTGTTTTAAATGATAACT	Cy5-CTTTCACGTCTTGACAAAGTTGAGGCTGAAGTGCA-BHQ2	GTTGAGTCACATATGTCTGCAAACTTTG	203
	Assay 3	NSP3: T24I	GTGCAAGGTTACAAGAGTGTGAATATGAT	FAM-TGAGAAGTGCTCTGCCTATACAGTTGAACTCGGTACAGAAG BHQ1	GGTGTAAGTAATTCAGATACTGGTTGCAAAG	178
		NSP3: L1266I	GTAGGAGACATTATACTTAAACCAGCAAATAATACTA	VIC-CAGAAGAGGTTGGCCACACAGATCTAATGGCTGC BHQ1	GGGTTTTCAAACCTAATACTCTAGATAATTCATTAGG	150
		Spike: V213G	AATATATTCTAAGCACACGCCTATTAATTTTGG	Cy3-CGTGATCTCCCTCAGGGTTTTTCGGCTTTAGAACC-BHQ2	CACCAGGAGTCAAATAACTTCTATGTAAAGC	151
		Spike: R408S	GATTCATTTGTAATTAGAGGTGATGAAGTCTGC	Cy5-CGCTCCAGGGCAAACTGGAAAGATTGCTG-BHQ2	GGTAATTATAATTACCACCAACCTTAGAATCAAG	162

### Plasmid construction and RNA transcript preparation

The viral NSP1, NSP3, NSP6, and Spike genes were synthesized and separately inserted into a plasmid pEasy-T1 (TransGen Biotech, Beijing, China), linearized by digestion with restriction enzyme for RNA transcript preparation by using the RiboMAX™ Large Scale RNA production System (Promega, WI, USA) according to the manufacturer’s instructions. Concentration of the RNA transcript was measured by using NanoDrop (Thermo Fisher Scientific, MA, USA). Copy number of the transcript was calculated by using the following formula: copy number (copies/ml) = 6.02×10^23^ copy/mol × molarity (mol/L)/Molecular Weight (g/mol). The quantified RNA transcript was then 10-fold diluted with RNase-free water as templates for development of the allele-specific qRT-PCR assays.

### Formulation and procedures of the developed multiplex allele-specific qRT-PCR assays

Takara Onestep PrimeScript™ III RT-qPCR Mix (Takara, Dalian, China) was used to build the multiplex qRT-PCR assays. Each reaction mix consisted of 10 μl One Step PrimeScript™ III RT-qPCR Mix, 0.4 μl forward primer (10 μM), 0.4 μl reverse primer (10 μM), 0.4 μl probe (5 μM), 2 μl RNA template, supplemented with RNase free water to a final volume of 25 μl (the concentration of each set of primers & probe (0.05 μM to 0.5 μM for each primer, and 0.025 μM to 2.5 μM for each probe) was evaluated in advance to reach an optimal work concentration). Each reaction was run in triplicate with 96-well plates by using a Roche LightCycler 480 (Roche, IN, USA). The thermocycling conditions for each qRT-PCR assay were as follows: 52°C 15 min for reverse transcription, 95°C 10 sec for thermal inactivation of the reverse transcriptase, followed by 45 cycles of 95°C 30 sec and 60°C 1min for PCR amplification (a thermal gradient experiment (58°C-62°C) was performed in advance to reach an optimal work temperature for annealing and extension). Fluorescence acquisition was set at the annealing/extension step of each cycle.

### Methodological evaluations of the developed multiplex allele-specific qRT-PCR assays

To determine the analytical sensitivity of the developed allele-specific multiplex qRT-PCR assays, we used the above mentioned *in vitro* transcribed RNA of the viral genes as templates. The quantified RNA transcript was 2-fold serially diluted from 100 copies/μl to 0.1 copies/μl, and subjected to each of the multiplex allele-specific qRT-PCR assay. Limit of detection (LoD, defined as the minimum detected concentration at 95% probability) for each qRT-PCR assay was determined through the Probit regression analysis implemented in SPSS v22.0 (IBM, IL, USA).

Linear detection range and amplification efficiency of the multiplex allele-specific qRT-PCR assay were determined by using 10-fold serially diluted RNA transcript of the viral genes as templates. Fitting curves generating from the quantity of RNA transcripts and the corresponding Cp values were applied for determination of the linear detection ranges of the qRT-PCR assays. Correlation coefficient (R^2^) in the fitting curve (ranging from 0-1, with the value closer to 1 suggesting higher linearity) was applied for evaluation of the detection linearity, with the threshold for good linearity being defined as R^2^ > 0.99. Amplification efficiencies of the qRT-PCR assays were evaluated by calculating the slope of the fitting curve, and defined as 10 ^(-1/slope)^-1. The value of amplification efficiency was ranged from 0 to 1, with a value closer to 1 indicating a higher amplification efficiency.

Reproducibility of the multiplex allele-specific qRT-PCR assays were evaluated by using the parameter “Coefficient of Variation (CV)”, which was calculated by the standard deviation of a Cp value for an RNA dilution divided by the men Cp values for all replicates of the same RNA dilution. Intra-assay and inter-assay reproducibility were assessed by the results of three replicates in one run and the results of three runs in three days, respectively.

Specificity of the multiplex allele-specific qRT-PCR assays were evaluated by checking possible cross-reactivity between the developed qRT-PCR assays and the nucleic acids from common respiratory viruses (including coronaviruses (NL63, OC43 and 229E), influenza viruses (A and B), parainfluenza viruses (types 1-4), and respiratory syncytial virus) and respiratory bacteria (including *Streptococcus pneumoniae*, *Haemophilus influenzae*, *Staphylococcus aureus* and *Pseudomonas aeruginosa*).

### Clinical performance of the developed multiplex allele-specific qRT-PCR assays

To further evaluate the performance of the developed multiplex allele-specific qRT-PCR assays, we conducted assay validation with a total of 105 nasopharyngeal swabs. SARS-CoV-2 nucleic acid detection was performed by using a previously reported assay (Liu et al., 2020; Xiao et al., 2021), and confirmed by Sanger sequencing. A volume of 20 μl RNA was extracted from 200 μl of each nasopharyngeal swab containing viral transport medium by using the Trizol RNA extraction method (Rio et al., 2010). Assay validation was performed by using 2 μl of the extracted RNA as template for each 20 μl qRT-PCR reaction system.

### Statistical analysis

All qRT-PCR experiments were performed in triplicate, and data are expressed as mean ± s.d. (the standard deviation). Fitting curves between the quantity of RNA transcript and the corresponding Cp values were generated by using Excel^®^ Microsoft^®^ 365. The amplification efficiency of a qRT-PCR assay was defined as 10 ^(-1/slope)^-1, whereas “slope” represents the slope of the fitting curve. R^2^ of the fitting curve was applied for description of the correlation coefficient between the quantity of RNA transcripts and the corresponding Cp values. The LoDs of the developed qRT-PCR assays were determined by using the Probit regression analysis module implemented in SPSS 22.0 at the 95% probability level. The kappa index evaluating the agreement between results of Sanger sequencing and the developed qRT-PCR assay was calculated by using the Kappa and the McNemar crosstab statistics implemented in SPSS 22.0.

## Results

### Description and result interpretation of the developed multiplex allele-specific qRT-PCR assays

**Figure 1:**
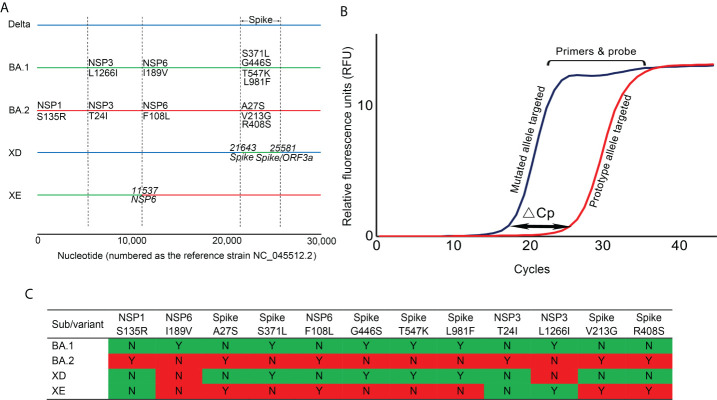
The principle and covered viral mutations of the developed multiplex allele-specific qRT-PCR assays. **(A)** The viral genomic locations of the 12 targeted mutations in the developed multiplex allele-specific qRT-PCR assays are shown, including 6 unique mutations in Omicron BA.1 subvariants (NSP3: L1266I, NSP6 I189V, Spike: S371L, Spike G446S, Spike T547K, Spike: L981F), 6 unique mutations in Omicron BA.2 subvariants (NSP1: S135R, NSP6: F108L, Spike A27S, Spike V213G, Spike R408s). The horizontal solid color lines represent the genome of the variant and subvariants of SARS-CoV-2. The vertical dashed lines indicate the boundary of viral genes. **(B)** The principle of the developed assays. The allele-specific qRT-PCR assays consist of two parallel reactions targeting the prototype allele and the mutated allele of each mutation, respectively. The inaccurate matched primers & probe to viral RNA generates a higher Cp value, while the accurate matched primers & probe to viral RNA generates a lower Cp value. The variation of Cp values (ΔCp) between the prototype allele targeting and the mutate allele targeting reactions was employed to determine if a mutation occurred. **(C)** A total of 12 mutations were enrolled in the developed assays, with 4 unique mutation patterns to identify the Omicron subvariants BA.1 and BA.2, and the recombinant variants XD and XE of SARS-CoV-2. The mutation pattern for the Omicron subvariants BA.1 and BA.2 were colored in green and red, respectively, those for the recombinant variants XD and XE were colored accordingly.

A panel of 3 multiplex allele-specific qRT-PCR assays targeting 12 key mutations (**Figure 1A**) were developed for differentiation of the SARS-CoV-2 XD and XE recombinant variants and the Omicron BA.1 and BA.2 subvariants (**Table 1**). Each assay contained two parallel reactions targeting the prototype allele and the corresponding mutated allele of each mutation, respectively. Each reaction contained 4 TaqMan hydrolysis probes targeting 4 mutations, labeled with 5’-FAM-BHQ1-3’, 5’-VIC-BHQ1-3’, 5’-CY3-BHQ2-3’, and 5’-CY5-BHQ2-3’, respectively. The Forward primer was designed to be allele specific, with one additional mutation at the 3’ terminus to amplify the mismatch effect. The variation of Cp values (ΔCp) between the two parallel reactions induced by primer-target mismatch was employed to differentiate the prototype allele and the mutated allele (**Figure 1B**). A mutation was determined to occur if the Cp value in the mutated allele targeting reaction was smaller than that in the prototype allele targeting reaction. The threshold was set as ΔCp > 2 according to the performance of all the three developed assays. If ΔCp ≤ 2, a repeated test is required to confirm the result. Viral variant was identified according to the obtained unique mutation patterns of the Omicron subvariants and recombinant variants of SARS-CoV-2 (**Figure 1C**).

### Analytical sensitivities of the developed multiplex allele-specific qRT-PCR assays

**Figure 2:**
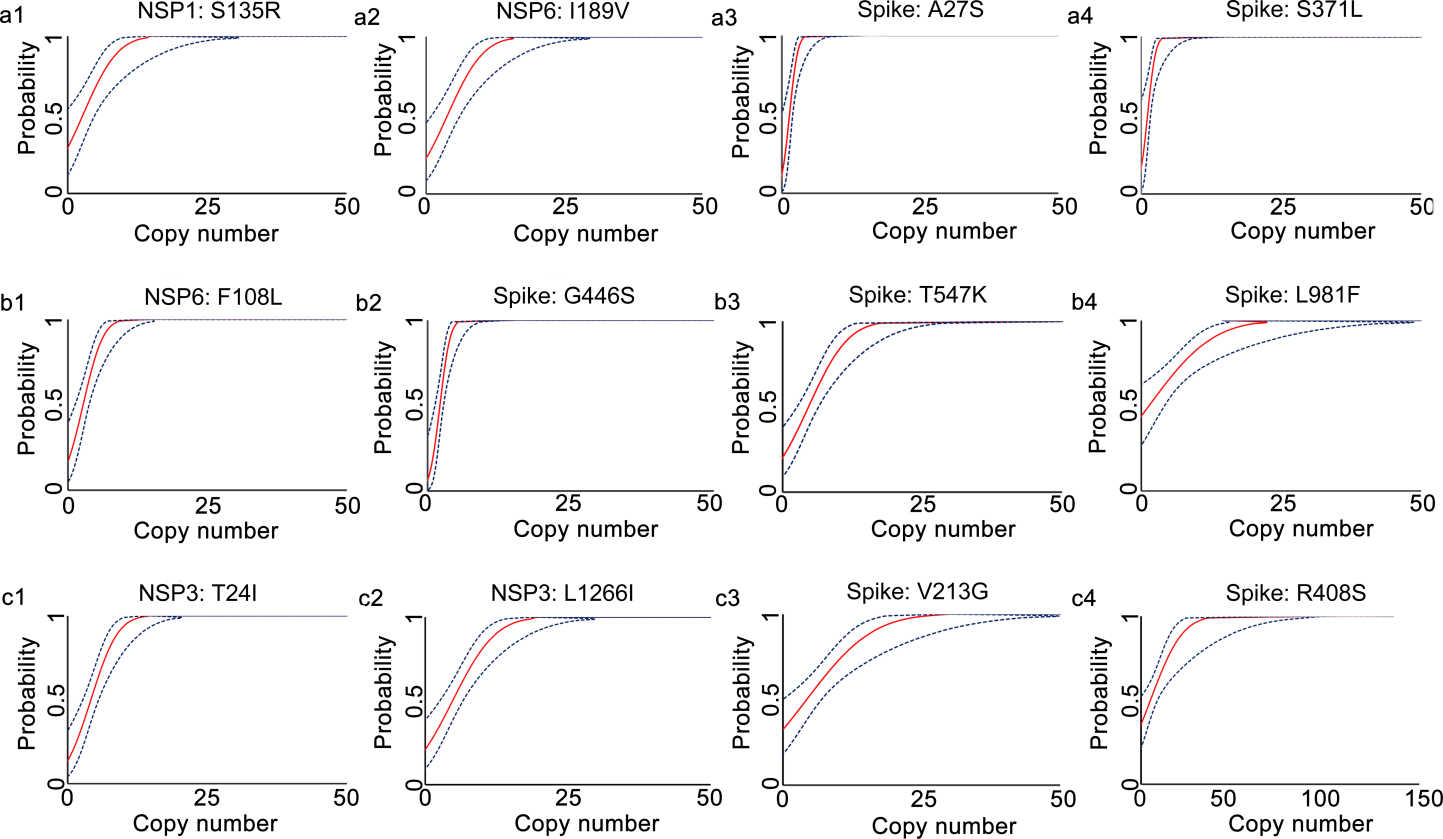
Determination of limits of detection for the developed multiplex allele-specific qRT-PCR assays to detect the prototype RNA of SARS-CoV-2. Limits of detection (LoDs) for the developed qRT-PCR assays to detect the prototype RNA of SARS-CoV-2 were determined by the Probit Regression (Dose-Response analysis) implemented in SPSS v22.0. qRT-PCR was performed by using the *in vitro* transcribed viral prototype RNA (2-fold dilution from 200 copies/reaction to 0.1 copies per reaction) as templates. The obtained Cp values and the corresponding copy number of viral RNA were applied for Probit Regression analysis. Probit curves for assay 1 [NSP1: S135R (a1), NSP6: I189V (a2), Spike: A27S (a3), and Spike: S371L (a4)], assay 2 [NSP6: F108L (b1), Spike: G446S (b2), Spike: T547K (b3) and Spike: L981F (b4)], assay 3 [NSP3: T24I (c1), NSP3: L1266I (c2), Spike: V213G (c3), and Spike: R408S (c4)] are shown. The inner solid line (in red) is a Probit curve. The outer dotted lines (in dark blue) are 95% CI.

**Figure 3:**
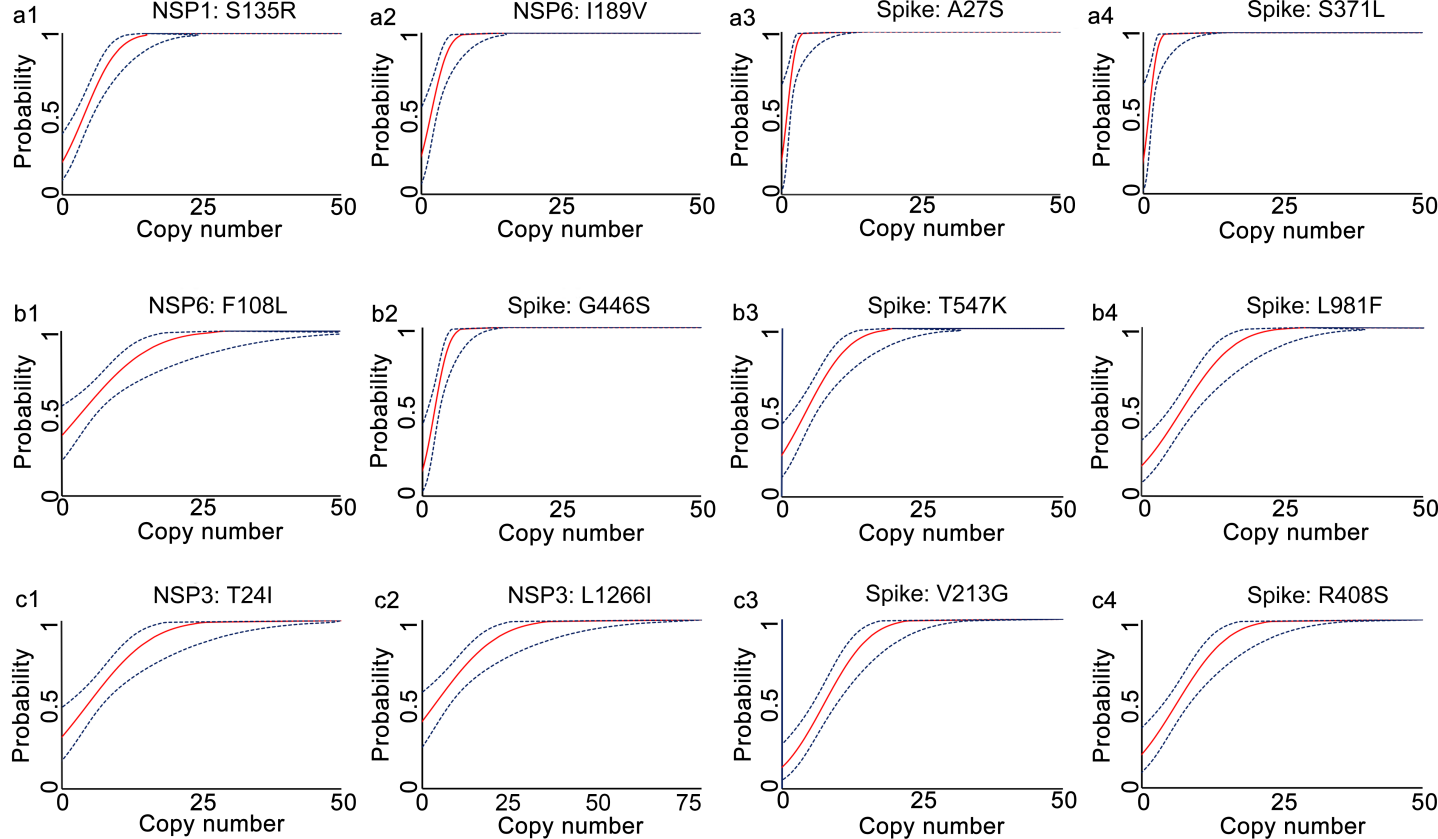
Determination of limits of detection for the developed multiplex allele-specific qRT-PCR assays to detect the Mutated RNA of SARS-CoV-2. Limits of detection (LoDs) for the developed qRT-PCR assays to the mutated RNA (covering all the alleles in **Table 1**) of SARS-CoV-2 were determined by the Probit Regression (Dose-Response analysis) implemented in SPSS v22.0. qRT-PCR was performed by using the *in vitro* transcribed mutated RNA (2-fold dilution from 200 copies/reaction to 0.1 copies per reaction) as templates. The obtained Cp values and the corresponding copy number of viral RNA were applied for Probit Regression analysis. Probit curves for assay 1 [NSP1: S135R (a1), NSP6: I189V (a2), Spike: A27S (a3), and Spike: S371L (a4)], assay 2 [NSP6: F108L (b1), Spike: G446S (b2), Spike: T547K (b3) and Spike: L981F (b4)], assay 3 [NSP3: T24I (c1), NSP3: L1266I (c2), Spike: V213G (c3), and Spike: R408S (c4)] are shown. The inner solid line (in red) is a Probit curve. The outer dotted lines (in dark blue) are 95% CI.

The analytical sensitivities of the developed qRT-PCR assays were represented with limit of detection (LoD), defined as the minimum detected template concentration at 95% probability (calculated through the Probit regression analysis). Using a 2-fold serially diluted *in vitro* transcribed RNA (from 200 to 0.1 copies per reaction) as templates, the LoDs for the prototype allele targeting reactions of the developed multiplex allele-specific qRT-PCR assays were determined to be 3.06-27.43 copies per reaction (**Table 2**, **Figure 2**), while the LoDs for the mutated allele targeting reactions were 2.97-23.65 copies per reaction (**Table 2**, **Figure 3**). These results suggested outstanding analytical sensitivities of the developed multiplex allele specific qRT-PCR assays.

**Table 2:** Linear detection ranges and amplification efficiencies of the developed multiplex allele-specific qRT-PCR assays.

Assay		Allele	Cp values (mean ± s.d.) for the 10-fold diluted *in vitro* transcribed prototype RNA template									R^2^	Slope	Efficiency	LoD (95% CI)
			10^7 *^	10^6^	10^5^	10^4^	10^3^	10^2^	10^1^	10^0^	10^-1^				
Prototype allele targeting reactions	Assay 1	NSP1: S135R	7.63 ± 0.01	11.72 ± 0.12	15.91 ± 0.05	21.21 ± 0.15	25.18 ± 0.03	29.25 ± 0.04	32.93 ± 0.17	36.65 ± 0.34	–	0.9978	-3.60	0.90	10.87 (7.76-21.95)
		NSP6: I189V	7.55 ± 0.01	11.99 ± 0.10	16.46 ± 0.09	22.02 ± 0.22	26.16 ± 0.01	30.39 ± 0.05	34.24 ± 0.25	38.08 ± 0.77	–	0.9974	-3.55	0.91	12.34 (9.12-21.88)
		Spike: A27S	7.79 ± 0.02	11.66 ± 0.08	15.65 ± 0.06	20.76 ± 0.17	24.66 ± 0.03	28.64 ± 0.03	32.16 ± 0.19	35.90 ± 0.53	39.38 ± 0.88	0.9981	-3.46	0.95	3.34 (2.61-6.16)
		Spike: S371L	8.57 ± 0.07	12.07 ± 0.33	16.95 ± 0.30	22.05 ± 0.26	25.55 ± 0.56	29.72 ± 0.52	33.71 ± 0.88	37.43 ± 0.99	–	0.9978	-3.66	0.88	3.06 (2.33-6.72)
	Assay 2	NSP6: F108L	8.87 ± 0.09	13.01 ± 0.41	18.63 ± 0.43	24.73 ± 0.38	29.22 ± 0.37	33.21 ± 0.52	36.18 ± 0.62	39.36 ± 1.10	–	0.9867	-3.58	0.90	7.42 (5.83-11.70)
		Spike: G446S	8.10 ± 0.06	11.45 ± 0.35	16.09 ± 0.33	21.00 ± 0.31	24.41 ± 0.59	28.56 ± 0.55	32.33 ± 0.90	35.79 ± 0.86	38.74 ± 1.13	0.9978	-3.62	0.89	4.48 (3.59-7.24)
		Spike: T547K	7.35 ± 0.06	11.20 ± 0.04	14.74 ± 0.08	18.54 ± 0.03	22.18 ± 0.04	25.84 ± 0.06	29.67 ± 0.02	34.02 ± 0.45	38.74 ± 1.12	0.9995	-3.46	0.95	13.88 (10.76-21.90)
		Spike: L981F	7.85 ± 0.14	12.05 ± 0.02	15.93 ± 0.05	20.71 ± 0.11	23.99 ± 0.05	27.58 ± 0.07	32.64 ± 0.05	35.76 ± 0.29	39.84 ± 0.28	0.9983	-3.48	0.94	16.21 (11.68-32.78)
	Assay 3	NSP3: T24I	7.92 ± 0.09	11.57 ± 0.02	15.01 ± 0.03	19.58 ± 0.09	22.60 ± 0.05	25.78 ± 0.05	31.15 ± 0.09	33.87 ± 0.21	37.63 ± 0.56	0.9970	-3.75	0.85	10.46 (8.31-15.70)
		NSP3: L1266I	7.90 ± 0.04	11.65 ± 0.09	16.66 ± 0.07	20.19 ± 0.48	24.44 ± 0.16	28.05 ± 0.09	33.69 ± 0.41	37.01 ± 1.13	38.53 ± 0.92	0.9980	-3.49	0.93	14.53 (11.36-22.31)
		Spike: V213G	8.00 ± 0.05	12.01 ± 0.10	17.27 ± 0.09	20.95 ± 0.54	25.42 ± 0.18	29.16 ± 0.07	35.01 ± 0.42	38.52 ± 1.28	–	0.9981	-3.68	0.87	19.70 (14.56-34.58)
		Spike: R408S	8.00 ± 0.04	11.51 ± 0.08	16.30 ± 0.13	19.66 ± 0.48	23.86 ± 0.12	27.40 ± 0.12	32.95 ± 0.42	35.98 ± 0.77	–	0.9977	-3.57	0.91	27.43 (19.03-63.19)
Assay		Allele	Cp values (mean ± s.d.) for the 10-fold diluted *in vitro* transcribed mutated RNA template									R^2^	Slope	Efficiency	LoD (95% CI)
			10^7 #^	10^6^	10^5^	10^4^	10^3^	10^2^	10^1^	10^0^	10^-1^				
Mutated allele targeting reactions	Assay 1	NSP1: S135R	6.83 ± 0.04	10.86 ± 0.03	14.49 ± 0.02	18.06 ± 0.04	21.97 ± 0.02	25.57 ± 0.03	29.28 ± 0.08	32.91 ± 0.39	35.11 ± 0.45	0.9979	-3.61	0.89	11.89 (9.25-18.27)
		NSP6: I189V	7.48 ± 0.04	11.38 ± 0.07	15.00 ± 0.04	18.69 ± 0.04	22.59 ± 0.01	26.13 ± 0.06	29.90 ± 0.06	33.6 ± 0.40	36.18 ± 0.48	0.9989	-3.65	0.88	5.76 (4.29-10.96)
		Spike: A27S	7.74 ± 0.05	11.41 ± 0.08	14.83 ± 0.05	18.33 ± 0.08	22.10 ± 0.02	25.66 ± 0.01	29.29 ± 0.05	33.03 ± 0.54	35.67 ± 0.48	0.9993	-3.58	0.90	3.00 (2.24-7.89)
		Spike: S371L	6.85 ± 0.11	10.70 ± 0.13	14.24 ± 0.02	18.04 ± 0.02	21.68 ± 0.03	25.34 ± 0.04	29.17 ± 0.08	32.52 ± 0.18	36.24 ± 0.90	0.9999	-3.67	0.87	2.97 (2.24-7.89)
	Assay 2	NSP6: F108L	7.82 ± 0.12	11.66 ± 0.02	15.35 ± 0.03	19.88 ± 0.07	23.05 ± 0.06	26.46 ± 0.08	31.23 ± 0.02	34.25 ± 0.27	38.70 ± 1.18	0.9989	-3.83	0.82	19.94 (14.43-39.16)
		Spike: G446S	8.42 ± 0.02	10.15 ± 0.17	14.74 ± 0.05	18.10 ± 0.04	21.82 ± 0.02	25.49 ± 0.03	29.13 ± 0.04	32.67 ± 0.14	36.15 ± 0.43	0.9977	-3.58	0.90	5.57 (4.34-9.58)
		Spike: T547K	6.86 ± 0.07	10.83 ± 0.06	14.15 ± 0.06	17.80 ± 0.01	21.34 ± 0.06	25.30 ± 0.03	28.92 ± 0.27	32.49 ± 0.16	35.02 ± 0.63	0.9988	-3.58	0.90	14.32 (10.97-23.37)
		Spike: L981F	7.55 ± 0.06	11.36 ± 0.12	14.74 ± 0.03	18.35 ± 0.03	21.91 ± 0.03	25.86 ± 0.04	29.53 ± 0.31	33.17 ± 0.14	35.92 ± 0.72	0.9993	-3.60	0.90	19.34 (14.98-30.17)
	Assay 3	NSP3: T24I	7.51 ± 0.05	10.99 ± 0.08	14.22 ± 0.04	17.76 ± 0.03	21.20 ± 0.03	25.09 ± 0.02	28.66 ± 0.30	32.33 ± 0.21	34.83 ± 0.29	0.9990	-3.49	0.93	19.81 (14.73-35.16)
		NSP3: L1266I	6.77 ± 0.18	10.61 ± 0.02	14.05 ± 0.06	17.63 ± 0.08	20.85 ± 0.20	25.12 ± 0.03	28.93 ± 0.03	32.75 ± 0.37	35.71 ± 0.38	0.9992	-3.66	0.88	23.65 (16.53-34.48)
		Spike: V213G	7.45 ± 0.19	11.13 ± 0.03	14.68 ± 0.07	18.18 ± 0.07	21.44 ± 0.26	25.76 ± 0.01	29.59 ± 0.07	33.45 ± 0.42	36.94 ± 0.16	0.9993	-3.71	0.86	18.19 (14.54-26.07)
		Spike: R408S	7.51 ± 0.07	10.81 ± 0.01	14.15 ± 0.07	17.64 ± 0.06	20.77 ± 0.24	25.00 ± 0.01	28.76 ± 0.04	32.54 ± 0.37	36.11 ± 0.31	0.9988	-3.60	0.90	18.28 (14.20-28.27)

*The started concentrations for the prototype genes of NSP1, NSP3, NSP6 and Spike were 2.33×10^7^, 4.45×10^7^, 3.10×10^7^, 6.82×10^7^ copies per reaction, respectively. # The started concentrations for the mutated genes of NSP1, NSP3, NSP6 and Spike were 8.59×10^7^, 8.83×10^7^, 6.92×10^7^, 2.45×10^7^ copies per reaction, respectively. Fitting curves between the quantity of RNA transcripts and the corresponding Cp values were generated by using Excel^®^ Microsoft^®^ 365. R^2^ of the fitting curve represented the correlation coefficient between the quantity of RNA transcripts and the corresponding Cp values. The amplification efficiency of a qRT-PCR assay was defined as 10 ^(-1/slope)^-1, in which slope is the slope of the fitting curve.

### Linear detection ranges and amplification efficiencies of the developed multiplex allele-specific qRT-PCR assays

Linear detection range serves as a key indicator of qRT-PCR performance, which is defined as the span of signal intensities that display a linear relationship between quantity of RNA transcript and corresponding Cp values. By using a 10-fold diluted *in vitro* transcribed RNA as templates, the linear detection ranges of the developed multiplex allele-specific qRT-PCR assays were determined with the parameter Coefficient of Correlation (R^2^, ranging from 0-1, the value closer to 1 represents stronger correlation). In the prototype allele targeting reactions (**Table 2**), the linear detection ranges were 10^7^-10^-1^ copies per reaction (R^2^ > 0.99) for the alleles of A27S (Spike), G446S (Spike), T547K (Spike), L981F (Spike), T24I (NSP3), and L1266I (NSP3), while the other prototype allele targeting reactions exhibited linear detection ranges of 10^7^-10^0^ copies per reaction (R^2^ > 0.99). Meanwhile, all the mutated allele targeting reactions exhibited linear detection ranges of 10^7^-10^-1^ copies per reaction (**Table 2**).

**Figure 4:**
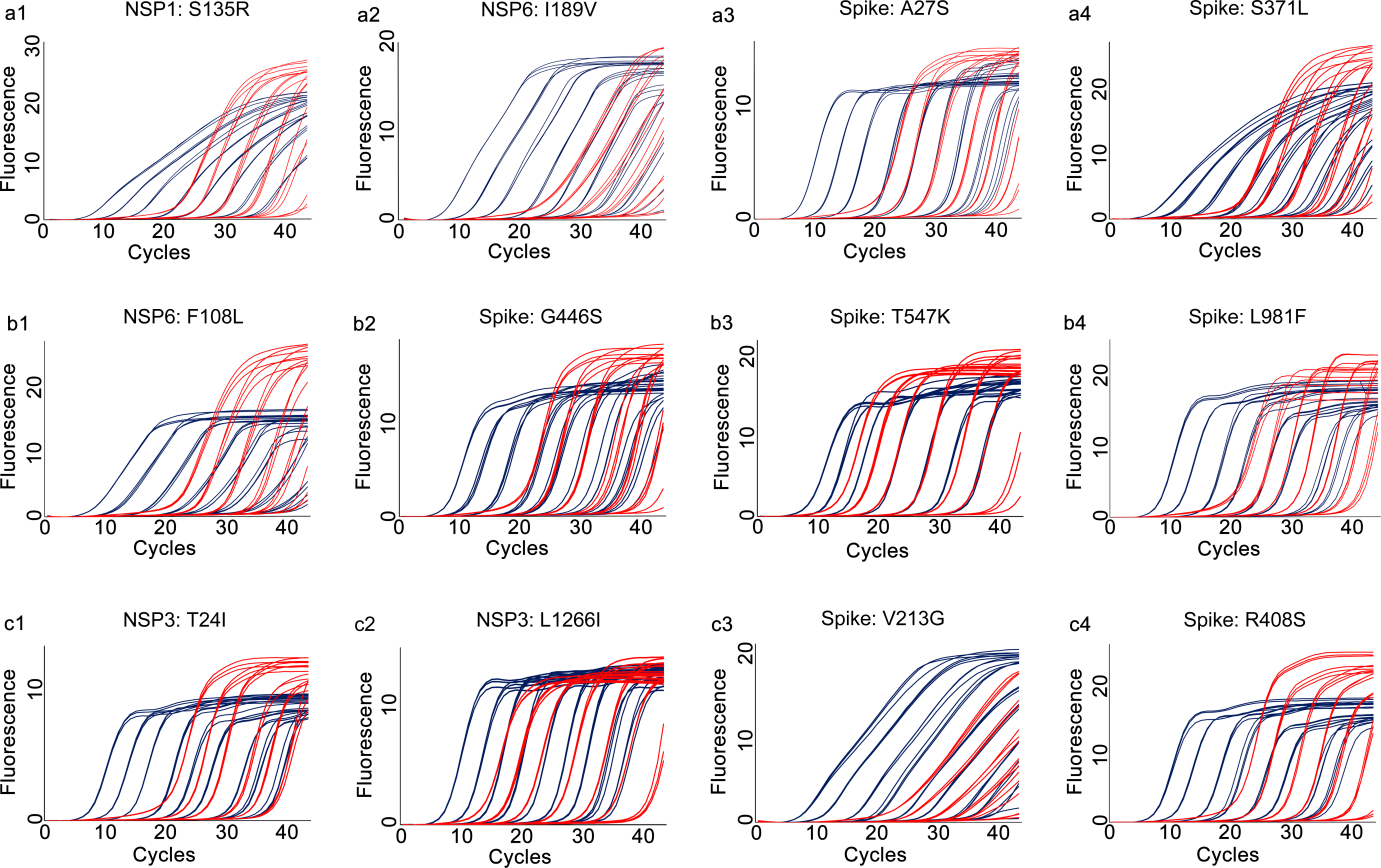
Amplification curves for the developed multiplex allele-specific qRT-PCR assays against the prototype genes of SARS-CoV-2. The viral prototype genes (NSP1, NSP3, NSP6 and Spike) were *in vitro* transcribed and 10-fold diluted from 10^7^ copies/reaction (2.33×10^7^, 4.45×10^7^, 3.10×10^7^, 6.82×10^7^ for NSP1, NSP3, NSP6 and Spike, respectively) to 10^-1^ copies/reaction. Two parallel reactions targeting the prototype allele and the mutated allele of the developed assays were performed by using the *in vitro* transcribed and serially diluted prototype RNA as template. The amplification curves for the prototype allele targeting reactions (in dark blue) were then merged with those of the corresponding mutated allele targeting reactions (in red). The amplification curves for assay 1 [NSP1: S135R (a1), NSP6: I189V (a2), Spike: A27S (a3), and Spike: S371L (a4)], assay 2 [NSP6: F108L (b1), Spike: G446S (b2), Spike: T547K (b3) and Spike: L981F (b4)], assay 3 [NSP3: T24I (c1), NSP3: L1266I (c2), Spike: V213G (c3), and Spike: R408S (c4)] are shown.

**Figure 5:**
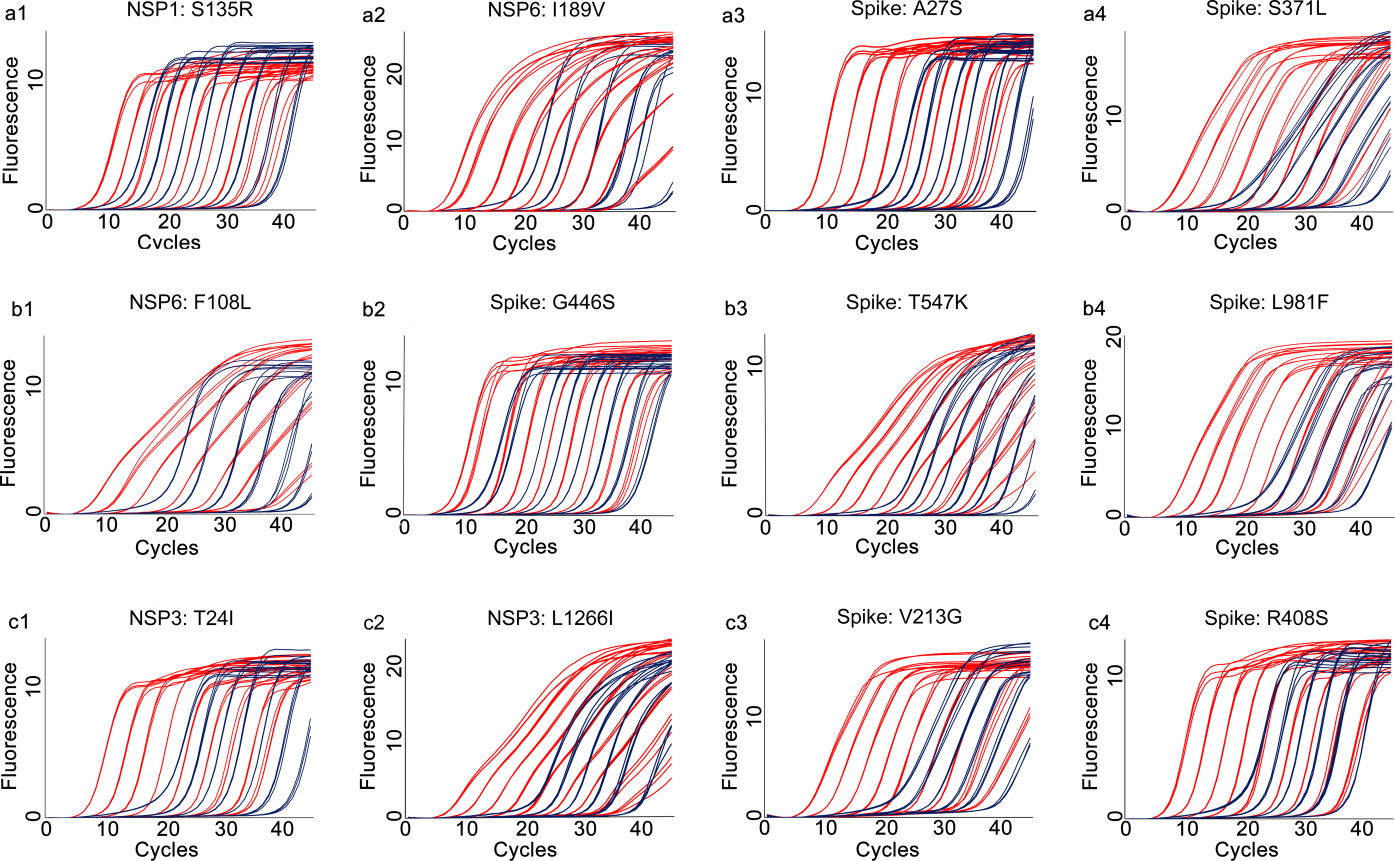
Amplification curves for the developed multiplex allele-specific qRT-PCR assays against the mutated genes of SARS-CoV-2. The viral mutated genes (NSP1, NSP3, NSP6 and Spike) covering all the mutations listed in **Table 1** were *in vitro* transcribed and 10-fold diluted from 10^7^ copies/reaction (8.59×10^7^, 8.83×10^7^, 6.92×10^7^, 2.45×10^7^ for NSP1, NSP3, NSP6 and Spike, respectively) to 10^-1^ copies/reaction. Two parallel reactions targeting the prototype allele and the mutated allele of the developed assays were performed by using the *in vitro* transcribed and serially diluted mutated RNA as template. The amplification curves for the mutated allele targeting reactions (in red) were then merged with those of the corresponding prototype allele targeting reactions (in dark blue). The amplification curves for assay 1 [NSP1: S135R (a1), NSP6: I189V (a2), Spike: A27S (a3), and Spike: S371L (a4)], assay 2 [NSP6: F108L (b1), Spike: G446S (b2), Spike: T547K (b3) and Spike: L981F (b4)], assay 3 [NSP3: T24I (c1), NSP3: L1266I (c2), Spike: V213G (c3), and Spike: R408S (c4)] are shown.

Amplification efficiency, defined as ratio of the actual amplicon number to the theoretical amplicon number from the same original template, with an efficiency value of 100% representing two copies of amplicon generated from one available template. We tested the amplification efficiencies of the developed assays by using the 10-fold diluted *in vitro* transcribed RNA as templates. The amplification curves of the prototype template (**Figure 4**) and the mutated template (**Figure 5**) exhibited approximately equal interval, indicating good amplification efficiencies of the developed assays. We further calculated the amplification efficiencies by using the formula: amplification efficiency = 10 ^(-1/slope)^ -1, in which the slope was from the fitting curve between the quantity of RNA transcript and the corresponding Cp values. The amplification efficiencies were ranged from 85% to 95% for the prototype allele targeting reactions, and ranged from 82% to 93% for the mutated allele targeting reactions (**Table 2**). These results demonstrated wide linear detection ranges and good amplification efficiencies of the developed multiplex allele-specific qRT-PCR assays for differentiation of SARS-CoV-2 recombinant variants (XD and XE) and the Omicron subvariants (BA.1 and BA.2).

### Reproducibility and specificities of the developed multiplex allele-specific qRT-PCR assays

The intra-assay and inter-assay reproducibility of the developed qRT-PCR assays were separately assessed by using Coefficient of Variation (CV) between quantity of RNA template and the corresponding Cp values in triplicate repeated tests. For the intra-assay reproducibility (**Table 3**), the CVs were 0.04-3.32 in the prototype allele targeting reactions and were 0.04-2.66 in the mutated allele targeting reactions. For the inter-assay reproducibility (**Table 4**), the CVs were 0.32-4.68 in the prototype allele targeting reactions and were 0.46-4.26 in the mutated allele targeting reactions. These results suggested good intra- and inter-assay reproducibility (with Coefficient of Variation less than 5%) of the developed multiplex allele-specific qRT-PCR assays for differentiation of SARS-CoV-2 recombinant variants and the Omicron BA.1 and BA.2 subvariants.

**Table 3:** Intra-assay reproducibility of the developed multiplex allele-specific qRT-PCR assays.

Assay			Allele	Coefficient of variation ^&^ (CV) of Cp Values for the 10-fold diluted *in vitro* transcribed prototype RNA template								
				10^7 *^	10^6^	10^5^	10^4^	10^3^	10^2^	10^1^	10^0^	10^-1^
Prototype allele targeting reactions	Assay 1	NSP1: S135R		0.13	1.02	0.31	0.71	0.12	0.14	0.52	0.93	–
		NSP6: I189V		0.13	0.83	0.55	1.00	0.04	0.16	0.73	2.02	–
		Spike: A27S		0.26	0.69	0.38	0.82	0.12	0.10	0.59	1.48	2.23
		Spike: S371L		0.82	2.73	1.77	1.18	2.19	1.75	2.61	2.64	–
	Assay 2	NSP6: F108L		1.01	3.15	2.31	1.54	1.27	1.57	1.71	2.79	–
		Spike: G446S		0.74	3.06	2.05	1.48	2.42	1.93	2.78	2.40	2.92
		Spike: T547K		0.82	0.36	0.54	0.16	0.18	0.23	0.07	1.32	2.89
		Spike: L981F		1.78	0.17	0.31	0.53	0.21	0.25	0.15	0.81	0.70
	Assay 3	NSP3: T24I		1.14	0.17	0.20	0.46	0.22	0.19	0.29	0.62	1.49
		NSP3: L1266I		0.51	0.77	0.42	2.38	0.65	0.32	1.22	3.05	2.39
		Spike: V213G		0.63	0.83	0.52	2.58	0.71	0.24	1.20	3.32	–
		Spike: R408S		0.50	0.70	0.80	2.44	0.50	0.44	1.27	2.14	–
**Assay**			**Allele**	**Coefficient of variation (CV) of Cp Values for the 10-fold diluted *in vitro* transcribed mutated RNA template**								
				**10^7 #^**	**10^6^**	**10^5^**	**10^4^**	**10^3^**	**10^2^**	**10^1^**	**10^0^**	**10^-1^**
Mutated allele targeting reactions	Assay 1	NSP1: S135R		0.59	0.28	0.14	0.22	0.09	0.12	0.27	1.19	1.28
		NSP6: I189V		0.53	0.62	0.27	0.21	0.04	0.23	0.20	1.19	1.33
		Spike: A27S		0.65	0.70	0.34	0.44	0.09	0.04	0.17	1.63	1.35
		Spike: S371L		1.61	1.21	0.14	0.11	0.14	0.16	0.27	0.55	2.48
	Assay 2	NSP6: F108L		1.53	0.17	0.20	0.35	0.26	0.30	0.06	0.79	3.05
		Spike: G446S		0.24	1.67	0.34	0.22	0.09	0.12	0.14	0.43	1.19
		Spike: T547K		1.02	0.55	0.42	0.06	0.28	0.12	0.93	0.49	1.80
		Spike: L981F		0.79	1.06	0.20	0.16	0.14	0.15	1.05	0.42	2.00
	Assay 3	NSP3: T24I		0.67	0.73	0.28	0.17	0.14	0.08	1.05	0.65	0.83
		NSP3: L1266I		2.66	0.19	0.43	0.45	0.96	0.12	0.10	1.13	1.06
		Spike: V213G		2.55	0.27	0.48	0.39	1.21	0.04	0.24	1.26	0.43
		Spike: R408S		0.93	0.09	0.49	0.34	1.16	0.04	0.14	1.14	0.86

^&^Coefficient of Variation, representing the reproducibility of the developed assay, was defined as the result of the standard deviation of Cp values divided by the mean Cp values of the triplicate qRT-PCR reactions using the same RNA template.

*The started concentrations for the prototype genes of NSP1, NSP3, NSP6 and Spike were 2.33×10^7^, 4.45×10^7^, 3.10×10^7^, 6.82×10^7^ copies per reaction respectively.

#The started concentrations for the mutated genes of NSP1, NSP3, NSP6 and Spike were 8.59×10^7^, 8.83×10^7^, 6.92×10^7^, 2.45×10^7^ copies per reaction, respectively.

**Table 4:** Inter-assay reproducibility of the developed multiplex allele-specific qRT-PCR assays.

Assay		Allele	Coefficient of variation ^&^ (CV) of Cp Values for the 10-fold diluted *in vitro* transcribed prototype RNA template								
			10^7 *^	10^6^	10^5^	10^4^	10^3^	10^2^	10^1^	10^0^	10^-1^
**Prototype allele targeting reactions**	Assay 1	Spike: S371L	1.32	1.24	0.68	1.14	0.39	0.52	1.23	2.55	–
		Spike: G446S	2.36	1.45	1.26	1.68	0.32	0.49	1.16	2.63	–
		Spike: T547K	0.65	1.03	1.68	1.36	0.88	0.68	0.95	2.39	2.91
		Spike: L981F	1.78	1.58	1.92	2.24	1.55	1.21	1.62	2.31	–
	Assay 2	Spike: A27S	0.84	2.69	1.12	1.88	2.01	2.58	1.68	2.38	–
		NSP6: F108L	0.97	2.53	1.89	2.11	1.66	2.35	2.46	3.25	3.61
		NSP6: I189V	1.29	1.68	2.12	1.68	1.90	1.35	0.87	1.66	3.97
		NSP1: S135R	1.44	1.65	3.57	2.87	1.30	1.56	2.86	3.44	2.65
	Assay 3	NSP3: T24I	0.98	0.62	1.26	2.05	2.58	3.65	1.52	2.41	3.02
		NSP3: L1266I	1.62	1.55	1.63	1.55	3.06	2.51	2.62	1.69	3.54
		Spike: V213G	2.52	1.68	1.29	3.67	1.29	2.57	2.66	4.02	–
		Spike: R408S	1.25	1.43	1.85	2.96	2.45	2.39	4.68	3.51	–
**Assay**		**Allele**	**Coefficient of variation (CV) of Cp Values for the 10-fold diluted *in vitro* transcribed mutated RNA template**								
			**10^7 #^**	**10^6^**	**10^5^**	**10^4^**	**10^3^**	**10^2^**	**10^1^**	**10^0^**	**10^-1^**
**Mutated allele targeting reactions**	Assay 1	Spike: S371L	2.36	2.59	0.68	0.79	0.88	0.68	1.28	2.68	3.87
		Spike: G446S	1.32	4.26	2.04	1.02	1.03	0.82	1.50	3.23	4.32
		Spike: T547K	2.03	1.25	1.25	0.46	1.48	0.47	0.96	2.54	4.08
		Spike: L981F	2.58	2.87	3.64	1.23	2.44	1.12	1.97	2.87	3.66
	Assay 2	Spike: A27S	1.78	1.12	0.58	1.52	1.31	1.35	1.25	3.35	3.82
		NSP6: F108L	1.36	2.99	0.96	2.96	2.16	1.88	2.33	2.45	1.93
		NSP6: I189V	2.65	2.38	1.88	1.12	1.82	0.92	3.76	4.03	1.76
		NSP1: S135R	2.14	2.71	1.32	1.67	1.73	0.69	3.28	3.25	2.56
	Assay 3	NSP3: T24I	1.32	1.16	1.53	1.31	0.82	0.77	3.43	1.73	2.08
		NSP3: L1266I	3.29	2.50	2.06	1.72	2.45	0.83	0.67	1.98	2.87
		Spike: V213G	2.68	1.36	1.61	1.43	3.26	0.58	0.87	2.67	3.24
		Spike: R408S	1.61	1.58	3.18	1.58	3.53	0.49	0.82	2.89	3.65

^&^Coefficient of variation, representing the reproducibility of the developed assay, was defined as the result of the standard deviation of Cp values divided by the mean Cp values of the triplicate qRT-PCR reactions using the same RNA template.

*The started concentrations for the prototype genes of NSP1, NSP3, NSP6 and Spike were 2.33×10^7^, 4.45×10^7^, 3.10×10^7^, 6.82×10^7^ copies per reaction respectively.

#The started concentrations for the mutated genes of NSP1, NSP3, NSP6 and Spike were 8.59×10^7^, 8.83×10^7^, 6.92×10^7^, 2.45×10^7^ copies per reaction, respectively.

**Figure 6:**
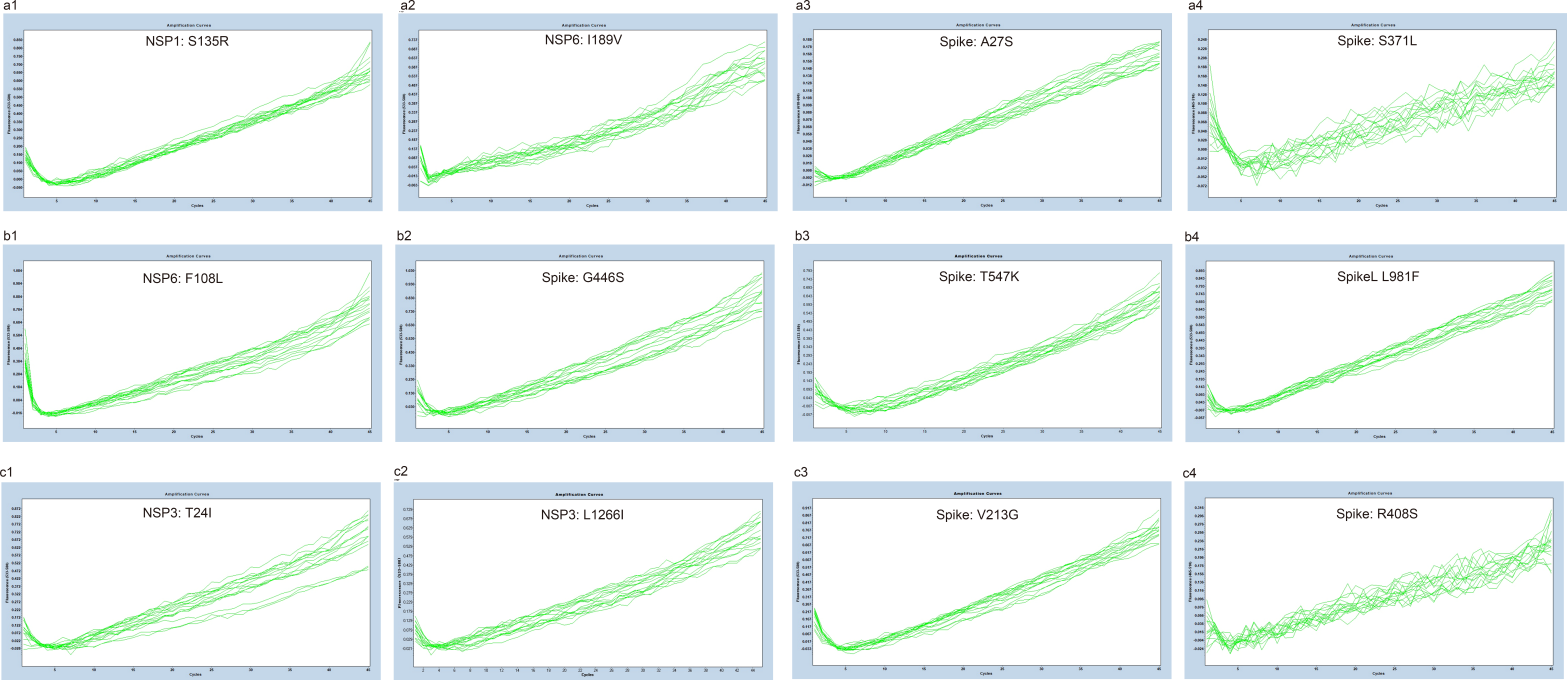
Evaluation of cross-reactivity between the developed assays and common respiratory viruses and pathogenic bacteria. The nucleic acids from the other coronaviruses (including NL63, OC43 and 229E), other respiratory viruses (including influenza viruses, parainfluenza viruses, and respiratory syncytial virus) and pathogenic bacteria (including *Streptococcus pneumoniae*, *Haemophilus influenzae*, *Staphylococcus aureus* and *Pseudomonas aeruginosa*) were used as templates (n=16) to check the cross-reactivity between the developed assays and common respiratory pathogens. The amplification curves for assay 1 [NSP1: S135R (a1), NSP6: I189V (a2), Spike: A27S (a3), and Spike: S371L (a4)], assay 2 [NSP6: F108L (b1), Spike: G446S (b2), Spike: T547K (b3) and Spike: L981F (b4)], assay 3 [NSP3: T24I (c1), NSP3: L1266I (c2), Spike: V213G (c3), and Spike: R408S (c4)] are shown.

No cross-reaction was observed between the developed qRT-PCR assays and other coronavirus (including NL63, OC43 and 229E), influenza viruses, parainfluenza viruses, respiratory syncytial virus, and common respiratory bacteria (**Figure 6**), suggesting good specificity of the developed multiplex allele-specific qRT-PCR assays.

### Clinical performance of the developed multiplex allele-specific qRT-PCR assays

A total of 105 clinical specimens were used to evaluate the clinical performance of the developed assays, which were tested for SARS-CoV-2 by a previously reported assay and confirmed by Sanger sequencing. Of the 105 clinical specimens (**Table 5**), 12 were Omicron BA.1 positive, 2 were Omicron BA.2 positive, 1 was variant XD positive, and 1 was variant XE positive by the developed assays, whereas 13 were Omicron BA.1 positive, 2 were Omicron BA.2 positive, 1 was variant XD positive, and 1 was variant XE positive by Sanger sequencing. Thus, the sensitivities of the developed assays against Sanger sequencing to detect the Omicron subvariants BA.1, BA.2 and the recombinant variants XD and XE were 92%, 100%, 100%, and 100%, respectively. The specificities of all the developed assays against Sanger sequencing to detect the Omicron subvariants BA.1, BA.2 and the recombinant variants XD and XE were 100%. The consistencies for the Omicron subvariants BA.1, BA.2 and the recombinant variants XD and XE between the developed assays and Sanger sequencing were 99.5% (Kappa=0.955, *P*>0.05), 100% (Kappa=1.000, *P*>0.05), 100% (Kappa=1.000, *P*>0.05), and 100% (Kappa=1.000, *P*>0.05), respectively. These results suggested good clinical performance of the developed multiplex allele-specific qRT-PCR assays.

**Table 5:** Clinical performance of the developed multiplex qRT-PCR assays for Omicron subvariants (BA.1 and BA.2) and recombinant variants (XD and XE) of SARS-CoV-2.

Omicron subvariant BA.1	Sanger sequencing		Performance characteristics						
	Positive	Negative	Sensitivity (%)	Specificity (%)	Youden’s Index	PPV (%)	NPV (%)	Agreement (%)	Kappa
The developed assay									
Positive	12	0	92.3	100	0.923	100	92.3	99.5	0.955 (*P*>0.05)
Negative	1	92							
Total	13	92							
Omicron subvariant BA.2	Sanger sequencing		Performance characteristics						
	Positive	Negative	Sensitivity (%)	Specificity (%)	Youden’s Index	PPV (%)	NPV (%)	Agreement	Kappa
The developed assay									
Positive	2	0	100	100	1.000	100	100	100	1.000 (*P*>0.05)
Negative	0	103							
Total	2	103							
Recombinant variant XD	Sanger sequencing		Performance characteristics						
	Positive	Negative	Sensitivity (%)	Specificity (%)	Youden’s Index	PPV (%)	NPV (%)	Agreement	Kappa
The developed assay									
Positive	1	0	100	100	1.00	100	100	100	1.000 (*P*>0.05)
Negative	0	104							
Total	1	104							
Recombinant variant XE	Sanger sequencing		Performance characteristics						
	Positive	Negative	Sensitivity (%)	Specificity (%)	Youden’s Index	PPV (%)	NPV (%)	Agreement	Kappa
The developed assay									
Positive	1	0	100	100	1.00	100	100	100	1.000 (*P*>0.05)
Negative	0	104							
Total	1	104							

Sensitivity =TP/(TP+FN) × 100%, specificity = TN/(TN+FP) × 100%, PPV (positive predictive value) = TP/(TP+FP) × 100%, NPV (negative predictive value) = TN/(TN+FN) × 100%, Agreement = (TP+TN)/(TP+FP+TN+FN) × 100%, whereas TP, TN, FP and FN represent the number of true positive, true negative, false positive, and false negative, respectively. Youden’s Index = (sensitivity + positivity)-1. Kappa index, assessing the agreement between Sanger sequencing and the developed multiplex qRT-PCR assays, was calculated by the Kappa and the McNemar crosstab statistics implemented in SPSS 22.0.

## Discussion

The desprepant transmissibility among Omicron subvariants and potential impacts of the recombinant variants of SARS-CoV-2 call for quick viral variant screening method to effectively monitor the viral transmission. Allele-specific qRT-PCR is an easy-to-perform and wide applicable method in SNP screening, representing a potential basic technology for developing viral variant screening assay. In the present study, we developed a panel of 3 multiplex allele-specific qRT-PCR assays targeting 12 key viral mutations for differentiation of SARS-CoV-2 recombinant variants (XD and XE) and the Omicron subvariants (BA.1 and BA.2).

Several technologies have been applied for screening of viral mutations. Sanger sequencing is one of the reliable methods to detect the rapidly emerging SARS-CoV-2 variants by providing a detailed view of mutations (Wang et al., 2021). Nevertheless, Sanger sequencing is time-consuming, a longer turnaround time to deal with, requiring expensive sequencer. Droplet digital RT-PCR also has been applied for screening of SARS-CoV-2 variants with the advantages of sensitive absolute quantification (Perchetti et al., 2021), but requiring costly apparatus. Next generation sequencing (NGS) based whole-genome sequencing (WGS) represents a promising strategy in searching viral mutations by providing a more comprehensive view of the viral genomic variations (Bull et al., 2020). However, the NGS based WGS costs higher and requires professional skills, making it hard for wide application especially in the developing areas. When facing the task of SARS-CoV-2 recombinant variant identification, the limitations of the above-mentioned technologies are multiplied, because multiple genomic regions are required to reach a clear conclusion.

qRT-PCR represents a promising approach in viral mutation screening with the advantages of time and cost effective, easy to manage, and wide applicable (Corman et al., 2020). qRT-PCR based short-amplicon high-resolution melting analysis (SA-HRM) was applied for SARS-CoV-2 variant screening, allowing cost-effective genotyping of single-nucleotide differences by the differential melting pattern of a short double-stranded DNA molecule (Diaz-Garcia et al., 2021) While SA-HRM is less specific and less robust than TaqMan assays, its results should be confirmed by TaqMan assays or DNA sequencing, making the overall processes time-consuming and costly.

Allele-specific qRT-PCR method provides a potential strategy in screening viral mutations, which has been widely used for SNP screening (Gaudet et al., 2009; Ngai et al., 2010; Cuenca et al., 2013). Although several allele-specific qRT-PCR assays have been developed for detecting SARS-CoV-2 variants either from clinical specimens (Borsova et al., 2021) or from waste water (Graber et al., 2021), a comprehensive assay covering the current circulating Omicron subvariants and the recent emerged recombinant variants of SARS-CoV-2 is lacking. To meet this demand, we developed and analytically validated a panel of 3 multiplex allele-specific qRT-PCR assays for differentiation of SARS-CoV-2 recombinant variants XD and XE, and the Omicron subvariants BA.1 and BA.2. The developed assays exhibited better or comparable analytical sensitivities with the previously reported allele-specific qRT-PCR assays for SARS-CoV-2 variants (Borsova et al., 2021; Wang et al., 2021; Garson et al., 2022). Based on the achievements of previous reports in detecting SNPs of West Nile virus (Worwa et al., 2014), genetic lineage of the infectious bursal disease virus (Tomas et al., 2017), recombination of infectious laryngotracheitis virus (Loncoman et al., 2017), and the developed assays for detecting SARS-CoV-2 Omicron subvariants and recombinant variants in the present study, we reached a conclusion that allele-specific qRT-PCR could be generalized as a methodology for quick differentiation of viral sublineages and recombination events.

As a potential shortcoming of the developed assays in this study, the unobserved or subsequent appearing mutations in the targeting area of the primers and probes could affect the performance of the assays by increasing the uncontrolled mismatch. To overcome this shortcoming, we select the unique mutations of the SARS-CoV-2 recombinant variants and the Omicron subvariants that are far away from the other existed viral mutations. Nevertheless, further evaluation is required to assess the potential influence of future mutations occurred in the targeting area of the primers and probes of the developed assay. Another potential shortcoming of the developed assays is possible disturbance of result interpretation by the intra-/inter- assay Cp variation between the prototype allele targeting reaction and the mutated allele targeting reaction. Because all the intra- or inter- assay Cp variations were no more than 5% (**Tables 3**, **4**) (that is to say, the highest variation of Cp ≤ 35×5% =1.75), we thus set the threshold as ΔCp > 2 to avoid results misinterpretation induced by intra-/inter-assay Cp variations.

In summary, a panel of 3 multiplex allele-specific qRT-PCR assays was developed for differentiation of SARS-CoV-2 recombinant variants and the Omicron subvariants. The developed assays exhibited outstanding analytical sensitivities (LoDs: 2.97-27.43 copies per reaction), wide linear detections ranges (from 10^7^ copies per reaction to 10^0^ copies per reaction), good amplification efficiencies (82% to 95%), good reproducibility (CVs < 5% in both intra-assay and inter-assay tests), and good specificity (not cross-reacted with other common respiratory viruses and bacteria). The developed assays provide an alternative tool for quick differentiation of SARS-CoV-2 recombinant variants XD and XE, and the Omicron BA.1 and BA.2 subvariants.

## Data Availability Statement

The original contributions presented in the study are included in the article/Supplementary Material. Further inquiries can be directed to the corresponding authors.

## Ethics statement

This study has been reviewed and approved by the Ethics Committee of Shanxi University. The patients/participants provided their written informed consent to participate in this study.

## Author contributions

JL and CW contributed to conception and design of the study. ZG, JC, RC, JN, JZ, YY, and XY performed the laboratory work. JC, GM, HL, and YD recruited participants to the study. JL and JX performed the statistical analysis. JL wrote the first draft of the manuscript. All authors contributed to manuscript revision, read, and approved the submitted version.

## Funding

This study has been funded by the Key R & D project of Shanxi Province (202102130501001 to JL, 202003D31005/GZ to CW, 202003D31003/GZ to JL).

## Acknowledgments

We thank Prof. Eric Leroy from University of Montpellier and Prof. Eric Westhof from University of Strasbourg for their valuable suggestions on the conception and design of the study, and critical comments on the draft of the manuscript.

## Conflict of interest

Authors GM and HL were employed by Shanxi Guoxin Caregeno Biotechnology Co., Ltd, Taiyuan, China.

The remaining authors declare that the research was conducted in the absence of any commercial or financial relationships that could be construed as a potential conflict of interest.

## Publisher’s note

All claims expressed in this article are solely those of the authors and do not necessarily represent those of their affiliated organizations, or those of the publisher, the editors and the reviewers. Any product that may be evaluated in this article, or claim that may be made by its manufacturer, is not guaranteed or endorsed by the publisher.
